# Bone marrow involvement in solid tumors - a retrospective observational study

**DOI:** 10.3389/fonc.2024.1434709

**Published:** 2025-09-08

**Authors:** Bicheng Zhang, Lan Jin, Dang Wu, Jing Xu, Siyu Guo, Jingjing Li, Ting Zhang

**Affiliations:** ^1^ Department of Radiation Oncology, The Second Affiliated Hospital of Zhejiang University, School of Medicine, Hangzhou, Zhejiang, China; ^2^ Department of Laboratory Medicine, The Second Affiliated Hospital of Zhejiang University, School of Medicine, Hangzhou, Zhejiang, China; ^3^ Department of Oncology, The Second Affiliated Hospital of Anhui Medical University, Hefei, Anhui, China

**Keywords:** overall survival, solid tumors (ST), bone marrow involvement, thrombocytopenia, bone marrow aspiration and biopsy

## Abstract

**Background:**

Solid tumor patients with bone marrow involvement (BMI) typically have a poor prognosis. However, some patients still exhibit a relatively good prognosis following aggressive treatment. This study aimed to identify prognostic factors to stratify patients and screen those who may benefit from aggressive treatment.

**Methods:**

A total of 41 consecutive patients diagnosed with a bone marrow biopsy were enrolled between January 1, 2016, and December 31, 2021. Then, their clinical features and laboratory data were assessed using the Chi-square test. Overall survival (OS) rates were plotted over time using the Kaplan-Meier method. Lastly, the survival time of solid tumor patients with BMI with distinct clinical symptoms, peripheral hematologic profiles, biochemical profiles, and coagulation function were analyzed using the Kaplan-Meier method.

**Results:**

The median OS of the whole cohort was 23 days. Specifically, the median OS of patients (n = 40) receiving anti-tumor treatment (n = 12) and supportive care (n = 28) was 158 days and 13 days, respectively (P < 0.001). Interestingly, the prognosis of patients with BMI and normal leukocyte count, platelet count, glutamic-pyruvic transaminase (GPT) level, and plasma prothrombin time (PT), as well as lactate dehydrogenase (LDH) level less than twice the normal upper limit and alkaline phosphatase (AKP) less than five times the normal upper limit, was relatively favorable. Conversely, elevated levels of LDH, AKP, GPT, prolonged PT, abnormal leukocyte count, and thrombocytopenia were associated with a poor prognosis.

**Conclusions:**

Solid tumor patients with BMI can benefit from aggressive anti-tumor treatments. Additionally, early diagnosis of BMI in solid tumor patients, selection of patients with a potential good prognosis, and administering appropriate anti-tumor treatments are key factors in prolonging the survival time of BMI patients.

## 1 Introduction

Bone marrow involvement (BMI) is an event involving tumor cells or cell clusters detaching from the primary tumor, colonizing, and proliferating in the bone marrow tissue. Its incidence rate in solid tumors has been reported to range from 11% to 30.2%, which is less prevalent than that in hematologic tumors (1–6). In patients with recurrent or metastatic disease, the incidence of BMI in solid tumors increases to 44% (5–11). The diagnosis of BMI involves the detection of tumor cells in bone marrow tissue. At present, bone marrow aspiration and biopsy are considered the gold standard for diagnosing BMI. However, bone marrow aspiration is an unconventional and invasive examination, potentially leading to poor patient compliance and misdiagnosis. Therefore, the actual incidence of BMI may be even higher than that documented in previous studies.

Solid tumor patients with BMI generally have a poor prognosis due to their poor general condition and the lack of effective treatment regimens. Recent studies have inferred that patients undergoing aggressive anti-tumor treatment can achieve a relatively good prognosis (1, 2, 6, 11, 12). However, factors influencing the prognosis of these patients remain elusive. Thus, the current study retrospectively analyzed the clinical characteristics, treatment regimens, and survival data of BMI patients with solid tumors to identify prognostic factors and stratify patients according to risk factors in order to screen patients who may benefit from aggressive treatments.

## 2 Materials and methods

### 2.1 Patients

The clinical information of patients with solid tumors who underwent bone marrow aspiration and biopsy from January 1, 2016, to December 31, 2021, at The Second Affiliated Hospital of Zhejiang University School of Medicine was collected and analyzed. Finally, 41 patients diagnosed with solid tumors and BMI via marrow aspiration and biopsy were included in this study. Information such as patient demographics, primary tumor location, number of metastatic sites, clinical symptoms, peripheral hematologic and biochemical profiles, coagulation function, treatments received, interval between diagnoses of primary tumor and BMI, survival time, and most likely cause of death was collected. Clinical symptoms encompassed fever and bone pain. Peripheral hematologic profiles included hemoglobin level, leukocyte count, and platelet count. Biochemical profiles comprised the level of alkaline phosphatase (AKP), lactate dehydrogenase (LDH), glutamic-pyruvic transaminase (GPT), glutamic oxaloacetic transaminase (GOT), albumin (ALB) and C-reactive protein (CRP), whilst coagulation profiles included plasma prothrombin time (PT) and activated partial thromboplastin time (APTT). In survival analysis, the term “elevated LDH level” referred to an increase to twice the normal upper limit. Similarly, the term “elevated AKP level” was defined as an increase to five times the normal upper limit. As a traditional Chinese custom, most patients opted for home-based end-of-life care. Consequently, the exact cause of death could not be confirmed.

### 2.2 Bone marrow aspiration and biopsy

Bone marrow aspirations were performed in the posterior iliac crest using a disposable bone marrow puncture biopsy needle (SA Medical Technology Co., Ltd. Shanghai). Aspirate smears were air-dried and stained with Wright-Giemsa stain. Bone marrow biopsy specimens were simultaneously acquired from an adjacent area on the iliac crest. These biopsy samples were subsequently decalcified in phosphate buffer for 12 to 24 hours and sequentially fixed in formalin, paraffin-embedded, and stained with hematoxylin and eosin. BMI was defined as infiltration by non-hemopoeitic cells. All bone marrow aspiration and biopsy specimens were retrospectively reviewed by a single pathologist.

### 2.3 Follow-up and statistical analysis

The overall survival time was calculated from the date of bone marrow biopsy to the date of death. All the patients were followed up until death or until the termination of this study on January 1, 2022. Basic demographic data were expressed as n (%) for categorical variables and medians for continuous variables. Patients were divided into groups based on clinical symptoms, peripheral hematologic profiles, and serum biochemical profiles. Group comparisons were performed using the Pearson χ2 test and Mann-Whitney U test. Overall survival was determined using the Kaplan-Meier method. The study proposal was approved by the Institutional Review Board.

Statistical analyses were conducted using SPSS 23.0 software (SPSS Inc, Chicago, USA), with *p* < 0.05 considered statistically significant.

## 3 Results

A total of 41 patients were included in this study (**Table 1**). Their median was 54 years (range 19-78 years), with 65.9% of patients being male. The most common primary tumor location was cancers of unknown origin (8 patients, 19.5%), followed by lung (7 patients, 17.1%), soft tissue (6 patients, 14.6%), stomach (5 patients, 12.2%), nasopharynx (5 patients, 12.2%), breast (3 patients, 7.3%), pancreas (2 patients, 4.9%), esophagus (2 patients, 4.9%), prostate (2 patients, 4.9%), and colon (1 patient, 2.4%). The interval between the diagnosis of the primary tumor and BMI ranged from 0 to 7578 days, and 58.5% of patients were diagnosed within one week after the diagnosis of the primary tumor. Among the 41 patients, 38 had complete imaging records. The correlation between BMI and other metastatic sites is described in **Table 1**. The most common metastatic site was bone (81.6%), followed by non-regional lymph node (42.1%), liver (26.3%), lung (15.8%), central nervous system (5.3%), adrenal gland (5.3%), pleura (5.3%), and peritoneum (5.3%).

**Table 1:** Characteristics of patients with solid cancer and bone marrow metastases.

	No. of patients/total patients (%)
Age, median (range)	54 (19-78)
Male gender	27/41 (65.9%)
Primary tumor	
Unknown origin	8/41 (19.5%)
Lung	7/41 (17.1%)
Soft tissue	6/41 (14.6%)
Stomach	5/41 (12.2%)
Nasopharynx	5/41 (12.2%)
Breast	3/41 (7.3%)
Pancreas	2/41 (4.9%)
Esophagus	2/41 (4.9%)
Prostate	2/41 (4.9%)
Colon	1/41 (2.4%)
Other metastatic sites	
Bone metastasis	31/38 (81.6%)
Non-regional lymph node metastasis	16/38 (42.1%)
Liver metastasis	10/38 (26.3%)
Lung metastasis	6/38 (15.8%)
Cerebra metastasis	2/38 (5.3%)
Adrenal metastasis	2/38 (5.3%)
Pleural dissemination	2/38 (5.3%)
Peritoneal dissemination	2/38 (5.3%)

The clinical symptoms and laboratory findings at the time of diagnosis of BMI are detailed in **Table 2**. Bone pain and fever were present in 68.3% and 56.1% of solid tumor patients with BMI, respectively. Meanwhile, anemia (78%) was the most common abnormal peripheral hematologic finding. Notably, 53.7% of all patients manifested moderate-to-severe anemia. Leukocyte abnormality was observed in 34.1% of patients, including 9.8% of patients with leukopenia and 24.4% with leukocytosis. Thrombocytopenia was noted in 56.1% of patients. Importantly, 26.8% of patients manifested grade IV thrombocytopenia, defined as a platelet count lower than 25x109/L. At the same time, elevated levels of lactate dehydrogenase (LDH), glutamic-pyruvic transaminase (GPT), and glutamic oxaloacetic transaminase (GOT), as well as hypoalbuminemia, were detected in 82.9%, 34.1%, 61.0%, and 58.5% of patients, respectively. Six patients failed to undergo the serum alkaline phosphatase (AKP) test at the time of diagnosis of BMI. Among the remaining 35 patients, 28 patients (80%) had elevated AKP levels. Plasma prothrombin time (PT) and activated partial thromboplastin time (APTT) were measured in 38 patients. Among them, prolonged PT and APTT were found in 25 (65.8%) and 15 (39.5%) patients, respectively. Concerning abnormal coagulation function, 3 patients had missed the test at the time of BMI diagnosis.

**Table 2:** The clinical symptoms and laboratory findings of patients with solid cancer and BMI.

	No. of patients/total patients (%)
Clinical symptoms	
Bone pain	28/41 (68.3%)
Fever	23/41 (56.1%)
Peripheral hematologic profiles	
Anemia	32/41 (78.0)
90 g/dl ≤ Hemoglobin < 126 g/dL	10/41 (24.4%)
Hemoglobin < 90 g/dL	22/41 (53.7%)
Leukocyte abnormality	14/41 (34.1%)
Leukocyte count > 10 × 10^9^/L)	10/41 (24.4%)
Leukocyte count < 4 × 10^9^/L)	4/41 (9.8%)
Thrombocytopenia	23/41 (56.1%)
75 ×10^9^/L ≤ Platelet < 100 ×10^9^/L	2/41 (4.9%)
50 ×10^9^/L ≤ Platelet < 75 ×10^9^/L	6/41 (14.6%)
25 ×10^9^/L ≤ Platelet < 50 ×10^9^/L	4/41 (9.8%)
Platelet < 25 ×10^9^/L	11/41 (26.8%)
Biochemical profiles	
Elevated LDH	34/41 (82.9%)
1-2 folds of UNL	13/41 (31.7%)
2-5 folds of UNL	10/41 (24.4%)
5-10 folds of UNL	6/41 (14.6%)
> 10 folds of UNL	5/41 (12.2%)
Elevated AKP	28/35 (80 %)
1-2 folds of UNL	7/35 (20 %)
2-5 folds of UNL	11/35 (31.4 %)
5-10 folds of UNL	5/35 (12.3 %)
> 10 folds of UNL	5/35 (12.3 %)
Elevated GPT	14/41 (34.1%)
1-2 folds of UNL	12/41 (29.3%)
> 2 folds of UNL	2/41 (4.9%)
Elevated GOT	25/41 (61.0%)
1-2 folds of UNL	11/41 (26.8%)
> 2 folds of UNL	14/41 (34.1%)
Hypoalbuminemia	24/41 (58.5%)
Abnormal coagulation function	29/38 (76.3%)
Prolonged PT	25/38 (65.8%)
Prolonged APTT	15/38 (39.5%)

Among the 41 patients with BMI, twenty-nine (70.7%) received only best supportive care, whereas the remaining twelve patients (29.3%) underwent anti-tumor therapy. Chemotherapy was the only treatment for 9 (22%) patients; 1 (2.4%) patient received radiotherapy alone, whilst 2 (4.9%) patients received both chemotherapy and radiation.

**Figure 1:**
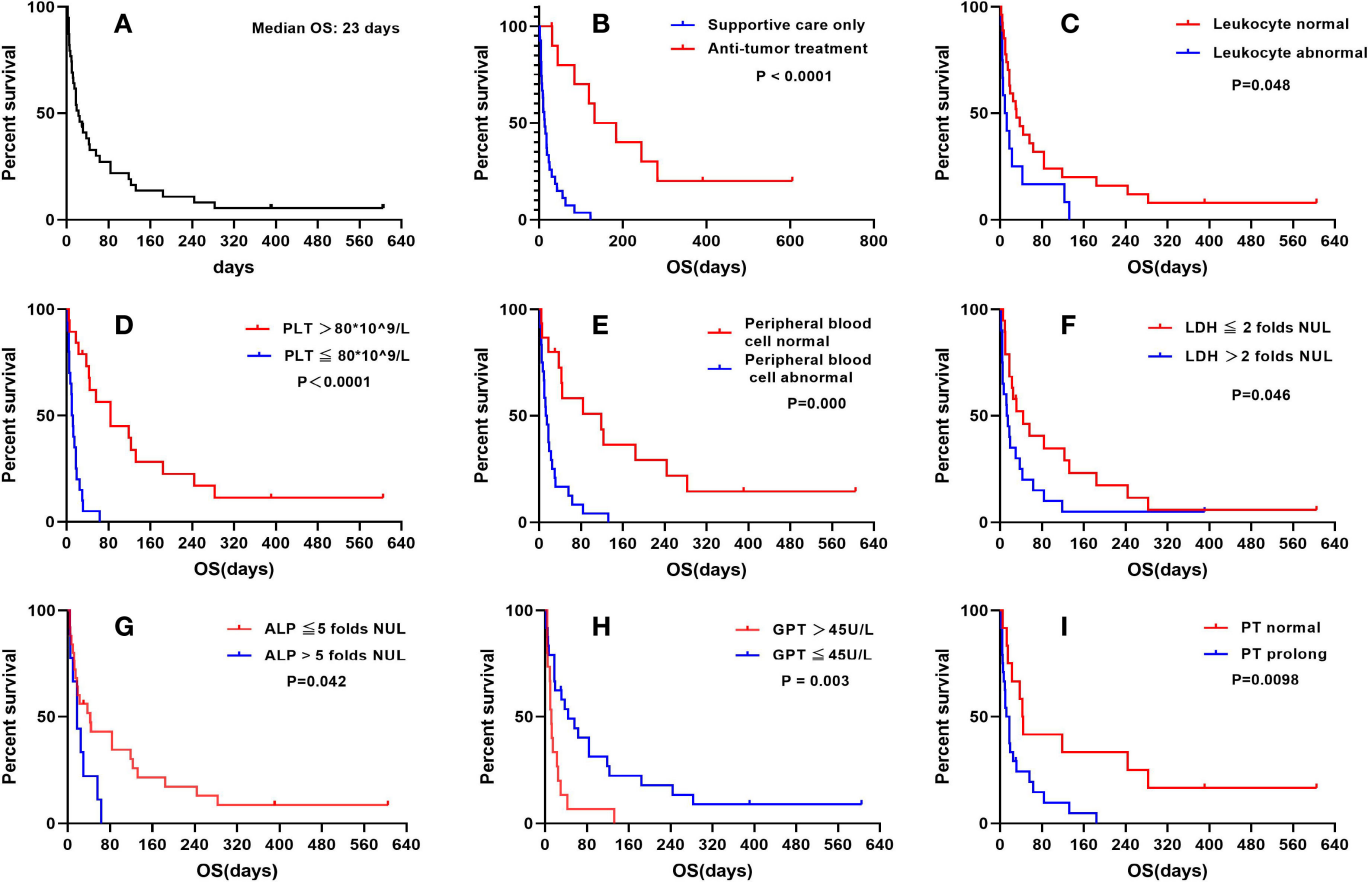
**(A)** The survival curves of solid tumor patients with BMI. **(B)** Survival curves of solid tumor patients with BMI receiving different treatments. **(C)** Survival curves of solid tumor patients with BMI with or without normal leukocyte counts. **(D)** Survival curves of solid tumor patients with BMI with different platelet counts. **(E)** Survival curves of solid tumor patients with BMI with or without normal peripheral blood cells. **(F)** Survival curves of solid tumor patients with BMI and different levels of LDH. **(G)** Survival curves of solid tumor patients with BMI and different levels of AKP. **(H)** Survival curves of solid tumor patients with BMI and different levels of GPT. **(I)** Survival curves of solid tumor patients with BMI and normal or prolonged PT.

Except for only three patients who survived until the end of follow-up (December 31, 2022), one patient was lost to follow-up, and all other patients have passed away. The median OS was 23 days among the 40 patients. The median OS of the patients who received anti-tumor treatment (n = 12) and supportive care (n = 28) was 158 days and 13 days, respectively (P < 0.001) (**Figure 1B**). Among the 12 patients who received anti-tumor treatment, the only patient who received radiotherapy survived for 119 days before succumbing to cerebral hernia. On the other hand, the median OS of patients who received chemotherapy (n = 9) was 84 days. One of them (soft tissue sarcoma) survived until the end of the study. Two patients (1 nasopharyngeal carcinoma and 1 neuroblastoma) who received both radiotherapy and chemotherapy survived until the conclusion of the study. However, 37 patients died, with 13 of them passing away within 10 days of BMI diagnosis. One patient was lost to follow-up after the diagnosis.

The results of survival analysis according to clinical symptoms and laboratory findings are summarized in **Table 3**. Neither fever nor bone pain was significantly correlated with the OS of BMI patients. Regarding peripheral hematologic profiles, the level of hemoglobin was also not associated with OS. However, the leukocyte and platelet counts were significantly correlated with a worse prognosis in BMI patients (**Figures 1C, D**). For biochemical profiles, the levels of LDH, AKP, and GPT were significantly associated with an unfavorable OS, whereas no significant correlations were identified between GOT and ALB levels with OS (**Figures 1F–H**). Concerning coagulation function, PT was significantly correlated with a poor prognosis, whereas APTT was not (**Figure 1I**).

**Table 3:** Analyses of survival according to the clinical variables.

Variables	Category	No. of patients	Median (days)	OS p value
Gender	Male Female	26 (65%) 14 (35%)	18 23	0.985
Fever	Yes No	23 (57.5%) 17 (42.5%)	18 44	0.306
Bone pain	Yes No	28 (70%) 12 (30%)	23 13	0.525
LDH	Exceeded 2 folds UNL Under 2 folds UNL	21 (52.5%) 19 (47.5%)	13 44	0.046
AKP	Exceeded 5 folds UNL Under 5 folds UNL	10 25	18 43	0.042
GOT	Exceeded 2 folds UNL Under 2 folds UNL	14 26	10 31	0.209
GPT	Normal Abnormal	24 16	40 10	0.003
ALB	Normal Abnormal	16 24	38 18	0.109
Leukocyte	Normal Abnormal	27 13	31 9	0.026
Hemoglobin	≥ 90 g/dL < 90 g/dL	17 23	30 19	0.111
Platelet	≥ 80 ×109/L < 80 ×109/L	20 20	84 10	<0.001
Peripheral blood cell	Normal	12	119	<0.001
PT	Abnormal Normal	28 12	12 43.5	0.0098
APTT	Prolong Normal Prolong	25 22 15	15 38 10	0.0845

UNL: upper normal limit.

Normal peripheral blood cells were defined as hemoglobin levels ≥ 90 g/dL and platelet counts ≥ 80 ×109/L, as displayed in **Figure 1E**. This criterion had a P value of less than 0.001 in the survival analyses. As anticipated, the p-value of thrombocytopenia was also lower than 0.001 in the survival analyses.

Taken together, patients with BMI receiving anti-tumor treatment had a longer survival time than those receiving supportive care only. Additionally, BMI patients with normal leukocyte and platelet counts, LDH levels below twice the normal upper limit, AKP below five times the normal upper limit, and normal GOT levels and PT generally exhibited a good prognosis.

## 4 Conclusions

BMI patients with normal leukocyte and platelet counts, LDH levels below twice the normal upper limit, AKP below five times the normal upper limit, and normal GOT levels and PT generally exhibited a good prognosis. Solid tumor patients with BMI can benefit from anti-tumor treatments. Early diagnosis of bone marrow involvement in solid tumor patients, coupled with the selection of patients with a favorable prognosis and the administration of suitable anti-tumor treatment, are key factors for prolonging the survival time of bone marrow-affected patients.

## 5 Discussion

Chemotherapy is regarded as the first-line treatment for patients with BMI. However, pancytopenia in patients with BMI is not a contraindication for chemotherapy. The uncertainty surrounding the impact of chemotherapy-induced myelosuppression on peripheral blood cytopenia in BMI patients limits the administration of chemotherapy.

Ingle reviewed 43 patients with metastatic breast cancer and assigned them to a BMI-positive group and a BMI-negative group, with groups undergoing chemotherapy. In the first three cycles, the average percentage of full-dose chemotherapy given to the BMI-positive group was relatively lower compared to the BMI-negative group but was similar in subsequent chemotherapy cycles. However, the incidence of delayed treatment was higher in the BMI-positive group, especially in the first cycle. Besides, the duration of chemotherapy cycles was similar in both groups. Patients in the bone marrow-positive group required more hematological support, such as red blood cells or platelets, during the first 6 cycles (46% vs 21%). However, there were no cases requiring component blood transfusion in either group after 6 cycles. Chemotherapy-associated toxicity is primarily observed in the initial cycles. As tumor response after chemotherapy progresses, the toxicity experienced by patients in the BMI-positive group tended to be consistent with that in the BMI-negative group (8). Bezwoda reviewed 27 small cell lung cancer patients with BMI and 106 patients without BMI and documented that the incidence of treatment-related hematologic toxicity and requirements for hematologic support were comparable throughout the chemotherapy course (2). The role of chemotherapy in patients with poor performance status (PS) and BMI leading to pancytopenia can not be substituted by supportive care. Gilles Freyer reported 5 cases of breast cancer patients with BMI who suffered from poor PS (WHO 2 or 3) and BMI due to severe pancytopenia. All patients received hormone therapy in conjunction with weekly low-dose chemotherapy. Except for one early death unrelated to treatment, the remaining cases demonstrated significant improvement in PS, pain relief, and rapid normalization of blood cell counts. The overall survival period ranged from 12 to 38 months in the 3 patients who achieved objective tumor responses (13). Kopp reviewed 22 cases of breast cancer with BMI and described that among the 20 patients who received chemotherapy, abnormal peripheral hemogram findings, including anemia in 77.3% of patients and thrombocytopenia in 40.9% of patients, did not present major challenges in the subsequent treatment course. Four cases required dose reductions during the first cycle of chemotherapy. In addition, chemotherapy cycles were postponed in two patients, and the doses of cytostatic agents were reduced due to unresolved cytopenia (12).

The survival outcomes of solid tumor patients with BMI depend on the response rate to treatment. Small-cell lung cancer has historically demonstrated a high chemotherapy response rate, with reported median survival times ranging from 9 weeks to 8 months in small-cell lung cancer patients with BMI who underwent chemotherapy (1, 2, 6). KOPP reported a median survival time of 19 months among 22 breast cancer patients with BMI, with 20 patients receiving chemotherapy (12). Rodriguez-Kraul reviewed 48 metastatic breast cancer patients with BMI and evinced that treatment response significantly influenced survival outcomes. The median survival of patients with progressive, stable disease and partial response was 2 months, 17 months, and 22 months, respectively. Regarding patients who achieved complete response, the median survival time was not reached at the last follow-up (11).

For solid tumor patients with poor chemotherapy response, the survival time is relatively short. Hung reported a median survival time of 49 days in 83 solid cancer patients with BMI. Among them, thirty-three (40%) patients received only supportive care. Of the remaining 50 patients (60%) who received antitumor therapy, only 24% achieved a partial response, 16% had stable disease, and the remaining 60% had progressive disease (14). Zen reported a median survival time of 16 days in five patients with nasopharyngeal carcinomas and BMI. Of note, 4 patients died within 23 days under supportive care, and 1 patient survived over 3 months under 2 cycles of chemotherapy (15). Herein, the median survival time was 23 days in 41 solid cancer patients with BMI. The majority of the patients (70.7%) with BMI solely received the best supportive care, largely attributed to the clinical recommendations of doctors. The median survival time was 158 days in patients with BMI undergoing anti-tumor therapy and 13 days in those receiving exclusively best supportive care.

A reduction in the counts of peripheral blood cells is the main laboratory indicator in patients with hematopoietic abnormalities in the bone marrow. Anemia was reported in 71.4% to 100% of patients with BMI (8, 12, 14, 15), thrombocytopenia was observed in 40% to 80% of patients (8, 12, 14–16), and leukopenia was noted in 18% to 27% of patients (8, 12, 14, 15). Hung reported that 31% of patients experienced an increase in leukocyte count, also known as leukemoid reaction (14). Meanwhile, 24.4% of patients experienced an increase in leukocyte count in this study, which indicates abnormal bone marrow hematopoietic function in patients. This phenomenon entails the replacement of normal hematopoietic tissue by a large number of eosinophilic granulocytes in patients with BMI (5). However, these eosinophilic granulocytes do not function as neutrophilic granulocytes, which reduces the patient’s resistance. Biochemical profiles revealed elevated LDH, AKP, GOT, and GPT levels in patients with BMI (6, 15–17). Simultaneously, LDH levels were over 500 IU/L (Exceeded 2 folds UNL) in 44% to 54% of patients with BMI (11, 16). Patients with varying levels of biochemical indicators have not been comprehensively analyzed in previously published literature. Herein, BMI patients with LDH levels lower than twice the normal upper limit, ALP levels less than five times the normal upper limit, and normal GOT levels exhibited a better prognosis. Similarly, BMI patients with normal PT exhibited a better prognosis.

Peripheral blood cytopenia can manifest in patients developing myelosuppression after anti-tumor therapy, as well as those with BMI. They had similar peripheral blood counts and medical histories. Both myelosuppression and BMI may co-exist in patients with multiple organ metastases who have previously received multiple cycles of chemotherapy. It is crucial to distinguish between myelosuppression and BMI, given that their respective treatments significantly differ. Anti-tumor therapy should be discontinued in patients with myelosuppression, whilst supportive care, such as bone marrow hematopoietic stimulation, must be initiated. Furthermore, patients with BMI need to be treated with individualized antitumor therapies.

Bone marrow aspiration and biopsy are the gold standard for the diagnosis of BMI in solid tumor patients. It should be performed if the patient experiences symptoms such as bone pain, sternum tenderness, fever of unknown origin, anemia, leukopenia, thrombocytopenia, or laboratory tests that show elevated levels of LDH, AKP, GOT, and GPT (6, 15–17). Experts recommend bone marrow aspiration and biopsy as a routine staging procedure in patients with relevant clinical features and abnormal laboratory findings (15).

The remission of symptoms and normalization of pre-treatment hematological abnormalities (anemia, neutropenia, or thrombocytopenia) are considered indicators of treatment response. However, patients with BMI frequently have concomitant metastasis in other organs. The effectiveness of chemotherapy can be determined by evaluating other evaluable metastases. Nonetheless, bone marrow biopsy, as a direct test to assess bone marrow condition, should be repeated during the treatment process.

## Data Availability

The raw data supporting the conclusions of this article will be made available by the authors, without undue reservation.
